# Current review of glucagon-like peptide 1 receptor agonists and risk of nonarteritic anterior ischemic optic neuropathy

**DOI:** 10.1210/jendso/bvag224

**Published:** 2026-10-01

**Authors:** Franklin Ruan, Bhargavee Gnana, Cindy Mei, Winnie Gong, Siddharth Limaye, Daniel Kim, Erica A Person

**Affiliations:** Carle Illinois College of Medicine, University of Illinois Urbana-Champaign, Urbana, IL 61801, USA; Carle Illinois College of Medicine, University of Illinois Urbana-Champaign, Urbana, IL 61801, USA; Carle Illinois College of Medicine, University of Illinois Urbana-Champaign, Urbana, IL 61801, USA; Carle Illinois College of Medicine, University of Illinois Urbana-Champaign, Urbana, IL 61801, USA; Carle Illinois College of Medicine, University of Illinois Urbana-Champaign, Urbana, IL 61801, USA; Shiley Eye Institute, UC San Diego Health, CA 92037, USA; Carle Illinois College of Medicine, University of Illinois Urbana-Champaign, Urbana, IL 61801, USA; Carle Illinois College of Medicine, University of Illinois Urbana-Champaign, Urbana, IL 61801, USA; Department of Ophthalmology, Christie Clinic, Champaign, IL 61820, USA

## Abstract

Emerging data has identified a potential association between glucagon-like peptide-1 receptor agonists (GLP-1RAs) and nonarteritic anterior ischemic optic neuropathy (NAION), a typically sudden, painless, and irreversible cause of optic nerve-mediated vision loss. This review synthesizes the current clinical, epidemiologic, and potential mechanism linking GLP-1RA–based therapies to NAION risk, informing balanced risk assessment and interdisciplinary clinical decision-making.

A structured PubMed search was performed using terms including “GLP-1 receptor agonist,” “semaglutide,” “liraglutide,” “tirzepatide,” and “nonarteritic anterior ischemic optic neuropathy,” in addition to NAION risk factors, including disc-at-risk anatomy, type 2 diabetes, obstructive sleep apnea, hypertension, hyperlipidemia, renal disease, and ischemic heart disease.

A small cup-to-disc ratio (“disc-at-risk”) was found to be the most robust anatomical predictor of NAION. Systemic microvascular disease (diabetes, hypertension, hyperlipidemia) and conditions such as obstructive sleep apnea and end-stage renal disease were also linked to increased susceptibility to optic nerve head ischemia. Although data across electronic health records, national registries, and pharmacovigilance analyses suggest a very low absolute incidence of NAION, the use of GLP-1RAs demonstrates a statistically significant association with NAION development, yielding relative risk estimates between 1.2 and 4.3.

At this time, the potential association between GLP-1RA therapy and NAION should be interpreted as a safety signal rather than a contraindication. Screening for disc-at-risk anatomy, consideration of prior NAION history, and patient counseling regarding acute visual symptoms may be warranted prior to initiation of therapy. As evidence evolves, collaboration between endocrinologists and ophthalmologists will be essential to support safe and effective patient care.

## Context

Glucagon-like peptide-1 receptor agonists (GLP-1RAs) have reshaped modern metabolic care. Once reserved for type 2 diabetes mellitus (T2DM), these drugs are now first-line or add-on therapies for obesity (1, 2) and cardiometabolic risk (3) reduction, delivering weight loss and glycemic improvements. Large cardiovascular and renal outcome trials (4, 5) have further cemented their role as disease-modifying agents rather than simple glucose-lowering drugs. This rapid expansion of clinical indications necessitates further exploration into the safety profile (6). As millions of patients start GLP-1RAs, many of whom are younger, metabolically healthier, or treated outside traditional healthcare systems (7), rare adverse events that were invisible in preapproval trials are beginning to surface. One of these clinically consequential adverse events is nonarteritic anterior ischemic optic neuropathy (NAION), a typically sudden, painless, and generally permanent cause of vision loss, with a low incidence of 2.3-10.2 per 100 000 over age 50 (8).

Nonarteritic anterior ischemic optic neuropathy is the second most common cause of irreversible optic nerve–related visual loss in adults, surpassed only by glaucoma (9). Although our understanding of the pathogenesis of NAION remains incomplete, the leading theory involves hypoperfusion of the short posterior ciliary arteries supplying the optic disc. This reduced perfusion leads to swelling of the optic nerve head, resulting in axonal compression and subsequent ganglion cell apoptosis (10). Paradoxically, many risk factors for NAION overlap with conditions targeted by GLP-1RA therapies, including hypertension (HTN), hyperlipidemia (HLD), diabetes mellitus (T2DM), vascular disease, and obstructive sleep apnea (OSA) (10, 11). Of importance, however, is the risk factor of optic disc anatomy; up to 60% to 80% of patients with NAION have a small cup-to-disc ratio, also known as “disc-at-risk” (10, 12).

Over the past 2 years, multiple independent datasets have reported a small but statistically significant association between GLP-1RA therapy and incident NAION; however, the strongest and most consistent signal has been observed with semaglutide (13, 14). Although absolute risk remains very low, the reproducibility of this adverse event across study designs has prompted renewed attention at the interface of endocrinology and neuro-ophthalmology. The evidence now spans multiple study types: large claims/electronic health record (EHR) cohorts, national registry analyses, retrospective reviews, pharmacovigilance disproportionality analyses, and narrative reviews that synthesize mechanistic plausibility and clinical implications. This mini-review synthesizes the current clinical, epidemiologic, and possible mechanism between GLP-1RA–based therapies and NAION risk factors, informing balanced risk assessment and interdisciplinary clinical decision-making.

## Evidence acquisition

Studies discussing the risk of NAION in cardiometabolic disease were identified through a structured search of PubMed using keywords that included a combination of “GLP-1 receptor agonist,” “semaglutide,” “liraglutide,” “tirzepatide,” “non-arteritic ischemic optic neuropathy,” “NAION,” and “ischemic optic neuropathy.” Risk factors include disc-at-risk, type 2 diabetes, OSA, HTN, HLD, renal disease, and ischemic heart disease. Articles were excluded if they did not explicitly reference risk factor(s) or did not address NAION in the title, abstract, or indexed terms.

## Results and discussion

Among all identified risk factors, optic disc anatomy is a significant predictor of NAION. A small cup-to-disc ratio (often referred to as a “disc-at-risk”) was found to be present in a majority of patients, with prevalence estimates ranging from approximately 60% to over 80% depending on age and comorbidities (15-17). This association is commonly attributed to the crowding phenomenon, in which a small or absent physiologic cup (typically with cup-to-disc ratio < 0.2-0.3) results in tightly packed nerve fibers within the scleral canal. In this constrained anatomical space, even modest ischemic edema can rapidly increase intra-axonal pressure, exacerbating secondary compartment syndrome and reinforcing a cycle of ischemic injury (18). Importantly, González Martín-Moro et al identified cup-to-disc ratio as the most informative disc configuration parameter for NAION risk (19).

Additional anatomical factors may further increase susceptibility. For instance, optic disc drusen occupies space within the prelaminar optic nerve head, intensifying axonal crowding near the lamina cribrosa and lowering the threshold for ischemic injury (20). Emerging evidence also suggests that reduced anterior chamber volume may predispose certain eyes to transient intraocular pressure fluctuations, potentially contributing to optic nerve hypoperfusion in vulnerable individuals (21).

There are also a number of associated vascular risk factors that have been explored across multiple cohorts and meta-analyses, many of which have been implicated in both initial NAION and fellow-eye involvement. Diabetes mellitus is one of the most prevalent and consistently identified—affecting approximately 30% to 65% of patients with NAION and conferring a 1.5- to 3-fold increased risk compared with nondiabetic individuals (HR = 3.06, 95% CI: 1.50-6.26, *P* = .002; pooled OR = 1.64, 95% CI: 1.17-2.30, *P* = .004) (15, 22, 23). Diabetes is thought to exacerbate optic nerve head ischemia through several mechanisms. Hyperglycemia promotes leukostasis, increasing the risk of capillary occlusion and reducing effective blood flow within the optic nerve head microcirculation. Another theory proposes that hyperglycemia increases oxidative stress and the accumulation of advanced glycation end products, which impair vascular autoregulation and alter hemodynamics (24).

Additional microvascular risk factors frequently associated with NAION include HTN and HLD, although the magnitude of risk varies across studies. Hypertension has been reported in approximately 40% to 80% of patients with NAION, and meta-analysis data suggest a modest but significant association, with a pooled relative risk of approximately 1.3 (11). Proposed mechanisms include impaired autoregulation of optic nerve head blood flow and vasospasm of the short posterior ciliary arteries, which may result in episodic hypoperfusion (25). Lastly, HLD is also commonly observed in NAION, affecting approximately 50% to 65% of patients across cohorts (OR = 3.3, 95% CI: 1.4-7.8; RR = 1.43, 95% CI: 1.26-1.62, *P* < .00001) (11, 26). Elevated lipid levels are thought to promote endothelial dysfunction, oxidative stress, and atherosclerotic changes in the short posterior ciliary arteries, compromising microvascular perfusion of the optic nerve head (26).

In addition to these vascular risk factors, episodic causes of hypotension, such as OSA and end-stage renal disease (ESRD), have also been associated with increased risk for NAION. For instance, meta-analyses and cohort studies report a 2-fold or greater increased risk for NAION in patients with OSA (11). In studies employing polysomnography, OSA prevalence has been reported as high as 70% to 75% in patients with NAION, and the risk for NAION increases with OSA severity (HR = 3.36; 95% CI, 1.17-9.69; *P* = .025) (23). Furthermore, patients nonadherent to continuous positive airway pressure treatment had increased risk for second eye involvement (HR = 5.54; 95% CI, 1.13-27.11; *P* = .04) (27). Several mechanisms have been proposed, including recurrent nocturnal hypoxia directly affecting the optic nerve (28). Additionally, ESRD has been associated with a 3-fold risk of NAION in population-based cohort data (adjusted HR = 3.12; 95% CI = 2.10-4.64), while a recent systematic review further characterizes dialysis-associated NAION as a distinct clinical entity with poor visual outcomes and frequent bilateral involvement (29, 30).

Recent studies have also shown that NAION risk has been elevated in patients taking GLP-1RAs. Across the emerging NAION–GLP-1RA literature, population-based outcome studies generally report small to moderate relative risk elevations on a background absolute incidence of 2.3 to 10.2 per 100 000 over age 50 and 0.54 per 100 000 for all age groups (8), as described in Table 1. Hathaway et al, in a claimed-linked cohort of adults with type 2 diabetes or obesity-prescribed semaglutide, observed adjusted hazard ratios for NAION on the order of 4.3 compared with non-GLP-1RA regimens. Hathaway et al noted cumulative incidence rates as high as 8.9% over 3 years for GLP-1 users; though the high absolute risk used in their study may have been due to their study cohort being originally from a major neuro-ophthalmology referral center (13). In a larger, multinational insurance reimbursement analysis, Cai et al and Tesfaye et al reported more modest hazard ratios, typically between about 1.8 and 2.0 with patients taking GLP-1RAs (31, 32) Complementing these cohort designs, Azab et al used FDA Adverse Event Reporting System data to show disproportionately high reporting odds of NAION with semaglutide compared with other drugs (OR, 11.36; 95% CI, 8.33-15.49) (33). This variation in the current literature shows how effect estimates vary by dataset and comparator. Abbass et al and Wang et al, who used a US-based EHR database platform, found that the risk of developing NAION was either not statistically significant or modest near 1.0 at each follow-up time (14, 34). Incidence is not available in Abbass et al's study, and Wang et al reported a 2-year cumulative incidence up to 0.04% for those taking semaglutide. Consistent with these attenuated findings from observational studies, Silverii et al analyzed 69 randomized controlled trials and did not find a significant increase in NAION with GLP-1RAs (OR, 1.53; 95% CI, 0.53-4.44). This null finding may be due to only 5 out of 69 RCTs reporting NAION and the rarity of NAION events, with incident rates of 5.6 and 3.0 per 100 000 person-years. These findings may suggest that an association may be small and remains uncertain, given the uncommon nature of NAION events. A further breakdown of these comparisons can be seen in Table 1.

**Table 1:** Selected summary of published evidence evaluating associations between GLP-1 receptor agonists/incretin receptor agonists and NAION or related optic nerve outcomes

Study/year	Design and population	Drug exposure and comparator	NAION/optic outcomes	Effect size/key result	Absolute Risk	Notes and limitations
Hathaway et al, 2024 (13)	Claims-linked cohort; adults with T2DM or obesity at a single academic center; NAION identified from local/EHR plus codes	Exposure: Semaglutide (for T2DM or weight loss) Comparator: Non–GLP-1 RA metabolic regimens	Incident NAION	Semaglutide associated with markedly increased NAION risk; HR ≈ 4.3 (95% CI, ∼1.6-11.3) in diabetes, higher in obesity cohorts	T2D cohort: GLP-1RA: 8.9%, 36-month cumulative incidence Comparator:1.8%, 36-month cumulative incidence Overweight cohort: Glp-1RA: 6.7%, 36-month cumulative incidence Comparator: 0.8%, 36-month cumulative incidence	Single-center; modest event counts; NAION largely code based; high absolute risk may be due to the population being from a major neuro-ophthalmology referral center
Cai et al, 2025 (31)	Multinational, active-comparator new-user cohort in large OHDSI databases; adults with T2D initiating semaglutide	Exposure: Semaglutide Comparator: Other GLP-1RAs and non–GLP-1 RA agents (eg, SGLT2i and DPP-4i)	Incident NAION (specific and broad definitions)	Meta-analytic HRs for new semaglutide users vs non–GLP-1 RA ranged ≈1.23 to 2.27 depending on comparator and NAION definition; absolute incidence on the order of 10 to 50 per 100 000 person-years	Semaglutide: 14.5 and 8.7 cases per 100 000 person-years depending on NAION definition Comparator rates varied by individual drug; no pooled comparator absolute rate	Large, multidatabase; robust confounder adjustment; NAION definition still code based; effect size smaller than the original single-center signal but directionally consistent
Tesfaye, et al, 2026 (32)	Nationwide registry, target-trial emulation; patients with T2DM initiating GLP-1RA vs SGLT2i	Exposure: GLP-1 RAs (class, including semaglutide) Comparator: SGLT2 inhibitors	Presumed NAION (claims-based) (14)	GLP-1RA initiation associated with ≈85% increased NAION risk vs SGLT2i; pooled HR ≈ 1.85 (95% CI, ∼1.3-2.6); meta-analysis of semaglutide data with prior studies HR ≈ 2.78 (95% CI, 1.39-5.56);	GLP-1RA: 0.61 per 1000 person-years Comparator: 0.33 per 1000 person-years	Very large, population-based; active-comparator design; NAION definition presumptive (no chart review); residual confounding by indication and vascular risk remains possible; absolute risk increment small
Abbass et al, 2025 (14)	Multicenter US EHR/claims cohort (TriNetX); >100 M patients; stratified by T2DM and obesity	Exposure: GLP-1 RAs Comparator: Matched non–GLP-1 RA users	Incident NAION/ischemic optic neuropathy	In matched T2DM cohorts, relative risks for NAION at 1, 3, and 5 years with semaglutide vs controls were ∼1.0, 0.84, and 0.70, respectively; cumulative NAION ≤ 0.07% over 5 years; overall no clear class-wide excess NAION risk	T2D cohort: Semaglutide: 0.039%, 1-year cumulative incidence GLP-RAs: 0.059%, 1-year cumulative incidence High-BMI cohort: Semaglutide: 0.038%, 2-year cumulative incidence Comparator incidences are not reported	Very large, broadly representative; suggests attenuated or absent signal in routine care; however, NAION is rare, code based, and subject to misclassification; may dilute early high-risk windows
Azab and Pasina, 2025 (33)	FDA Adverse Event Reporting System (FAERS) pharmacovigilance study	Exposure: Semaglutide Comparator: All other agents in FAERS	NAION/ischemic optic neuropathy reports	Disproportionately elevated reporting odds: 11.36 (95% CI, 8.33-15.49)	Cannot estimate incidence GLP-1RA was used in 2.9% of 1559 NAION cases	Spontaneous reports only; cannot estimate incidence or causality; subject to under-reporting, notoriety bias, and channeling; useful for hypothesis generation and signal detection only
Wang et al, 2025 (34)	Nationwide US TriNetX EHR cohort and other health systems; adults with T2DM	Exposure: Semaglutide or tirzepatide Comparator: Other glucose-lowering therapies (non–GLP-1 incretins)	NAION and broader optic nerve/visual pathway disorders	Modest increased hazards for NAION/optic pathway diagnoses with semaglutide/tirzepatide vs comparators; HR 1.76 (95% CI, 1.01-3.07)	Semaglutide: Comparator: 0.02%, 2-year cumulative incidence	Target-trial emulation with PS matching; broad diagnostic cluster increases sensitivity but may include miscoded or nonischemic optic events
Silverii et al, 2025 (35)	Meta-analysis of 69 randomized controlled trials; patients with diabetes or obesity	Exposure: Any GLP-1RA Comparator: Placebo or active treatment	Ischemic optic neuropathy	OR, 1.53 (95% CI, 0.53-4.44); not significant	GLP-1: 5.6 per 100 000 person-years Comparator: 3.0 per 100 000 person-years	NAION is a very rare event. Only 5 RTCs had ischemic optic neuropathy. There were only 8 events in the GLP cohort and 4 in the comparator cohort

As many of these early studies are observational in nature, residual confounding remains an important point of discussion. Hathaway et al and Abbass et al propensity matched patients according to the covariates of sex, age, cardiovascular disease burden (OSA, HTN, HLD, CAD, etc.), and concomitant medications (amiodarone, phosphodiesterase inhibitors), although characterization of diabetes duration and severity was notably missing in both analyses. Cai et al matched patients based on similar characteristics as Hathaway et al and Abbass et al, although with the addition of a Diabetes Comorbidity Severity Index score as well as a more general Charlson Comorbidity Index. Tesfaye et al utilized a matching system with an expansive number of variables, including variables for demographics, proxies of diabetes severity and complications, cardiovascular history, claims-based frailty index, ophthalmic history, therapies, and medications. In particular, ∼45% of participants also had information on hemoglobin A1c and estimated glomerular filtration rate. The study design in Azab et al using FAERS compares reporting frequencies and, unlike cohort studies, cannot meaningfully adjust for confounders. Silverii et al analyzed available randomized controlled trials, which have a lower risk of confounded results. Wang et al matched for social determinants of health, obesity, cardiovascular health, diabetes medications, and diabetes complications. However, more specific diabetes severity ratings were not given. Overall, most studies had strong study designs, though the nature of observational studies suggests that confounding by indication cannot be fully ruled out.

The established pathophysiology of NAION in current literature is largely attributed to well-documented risk factors, including optic disc crowding, systemic vascular risk factors, hypoperfusion, OSA, and ESRD (11, 23, 24). Distinct from these established mechanisms, we propose a hypothesis that GLP-1RA use may trigger NAION through the worsening of compartment syndrome from osmotic stress caused by rapid, comparative hypoglycemia. Semaglutide has been found to decrease HbA1c by 1.40% and fasting blood glucose by 1.99 mmol/L when compared to placebo (36). Following sudden decreases in glucose after GLP-1RA administration, extracellular osmolarity may decrease faster than intracellular osmolarity, leading to intracellular edema due to the osmotic gradient (37). This initial swelling can cause compression of venules feeding the central retinal vein, exacerbating edema and ischemia (38, 39). In a 2010 analysis of 20 060 patients (40), bilateral diabetic papillopathy occurred in 5 patients over 4.9 years; notably, all affected patients had small cup-to-disc ratios and HbA1c at a significantly higher rate than the study population (*P* < .001). Furthermore, a 2025 case series of ophthalmic complications (41) noted 2 patients with rapid drops in HbA1c from 8.4% to 6.3% and 10.2% to 5.5% over roughly 3 to 4 months. Both patients developed disc edema, with the latter having a small cup-to-disc ratio and the former developing irreversible vision loss from NAION. As such, 1 speculative hypothesis for NAION incidence following GLP-1RA use is the susceptibility of the optic nerve head to microvascular changes and edema (42). Further investigation is warranted to validate this hypothesis and explore potential links between them. While absolute risk of NAION remains extremely low, the current data reports a trend that warns that any increase in relative risk must be weighed against the rarity of NAION and the established cardiometabolic benefits of GLP-1RAs.

## Conclusions

Although a growing number of independent datasets now report an association between GLP-1RA use and incident NAION, the available evidence is driven predominantly by studies of semaglutide. The existing evidence remains insufficient to conclude that NAION represents a class effect shared by all GLP-1RAs, as evidence for other agents remains comparatively sparse. Observational studies from large EHR networks and pharmacovigilance datasets have demonstrated a small but statistically significant increase in NAION risk among GLP-1RA users (13, 34). Across these study designs, findings are limited by residual confounding factors, generalization of the optic disc anatomy, and incomplete clinical data. Furthermore, the studies may be influenced by surveillance bias due to potentially higher rates of medical evaluation in the GLP-1RA groups, increasing the rate at which NAION may be added to the patient's chart. However, the risk for surveillance bias is somewhat dampened by the nature of NAION being an acute pathology. In contrast to silent diseases that patients may be largely unaware of and are therefore often recognized during routine examinations, NAION, characterized by sudden significant vision loss, is often accompanied and diagnosed by a visit to the emergency department.

As such, the current association between GLP-1RA use and NAION should be interpreted at this time as a meaningful safety concern rather than a contraindication to therapy, particularly given the well-established metabolic and cardiovascular benefits of GLP-1RA–based therapies. Outside of T2DM and obesity, GLP-1RA medications demonstrate beneficial effects on conditions such as cardiovascular disease, chronic kidney disease, metabolic dysfunction-associated liver disease, osteoarthritis, and OSA. There can be strong overlap between areas of GLP-1RA usage and NAION risk, including T2DM, HTN, HLD, and OSA. Out of all risk factors, disc architecture is the strongest predictor of NAION, with 60% to 80% of patients with NAION demonstrating a small cup-to-disc ratio (14-16). Vascular factors are also significant; T2DM increases NAION risk up to 3-fold, while HTN and HLD increases risk up to 1.5-fold (14, 21, 22, 24, 25). Episodic causes of HTN, like OSA and ESRD, have been found to double the risk for NAION (24, 29, 30). Because NAION is a multifactorial disease process, each individual patient's comorbidities and risk factors should be taken into account when prescribing GLP-1RAs.

Given that no clinically proven treatment exists for NAION and that approximately three-fifths of patients with NAION fail to recover visual acuity (43), risk–benefit analysis and clear communications with patients are essential. Prior to initiating GLP-1RA therapy, patients should be informed of the rare but potentially irreversible risk of vision loss and instructed to seek immediate emergency evaluation for any acute, painless monocular visual loss. Rapid assessment is critical to both diagnose NAION and to distinguish it from other emergent causes, such as occipital stroke. In patients with multiple metabolic risk factors or other clinical features that may increase concern for NAION, clinicians should consider referral to eye care professionals for an assessment of the cup-to-disc ratio to rule out the possibility of a disc-at-risk. Furthermore, as all patients with diabetes are already recommended to receive a dilated eye exam annually, additional attention paid to the cup-to-disc ratio at the time of their regularly scheduled exam could help stratify patients into the lower risk category (with a larger cup-to-disc ratio), relieving concern for this rare but devastating complication of NAION. This recommendation is supported by guidance from the American Optometric Association Evidence-Based Optometry Committee (44, 45), which identifies patients over 50 years of age with a “disc-at-risk” as being at the greatest risk for GLP-1RA–associated NAION and emphasizes the importance of regular comprehensive dilated eye examinations in this population. Additionally, patients with history of NAION are not ideal candidates of GLP-1RA use due to the risk of developing NAION in the fellow eye.

As specific guidance regarding NAION risk and GLP-1RA use continues to evolve, these agents remain transformative therapies for metabolic disease. A balanced approach that integrates awareness of ocular risk, individualized assessment, and counseling ensures that patients can benefit from GLP-1RA therapy while making informed decisions about their care.

## Data Availability

No new data were generated or analyzed in this review study. All data and information discussed in this review are derived from previously published sources which are cited throughout the manuscript.
